# Circadian Phase Has Profound Effects on Differential Expression Analysis

**DOI:** 10.1371/journal.pone.0049853

**Published:** 2012-11-20

**Authors:** Polly Yingshan Hsu, Stacey L. Harmer

**Affiliations:** Department of Plant Biology, University of California Davis, Davis, California, United States of America; USDA-ARS, United States of America

## Abstract

Circadian rhythms are physiological and behavioral cycles with a period of approximately 24 hours that are generated by an endogenous clock, or oscillator. Found in diverse organisms, they are precisely controlled and provide growth and fitness benefits. Numerous microarray studies examining circadian control of gene expression have reported that a substantial fraction of the genomes of many organisms is clock-controlled. Here we show that a long-period mutant in Arabidopsis, *rve8-1,* has a global alteration in phase of all clock-controlled genes. After several days in constant environmental conditions, at which point the mutant and control plants have very different circadian phases, we found 1557 genes to be differentially expressed in *rve8-1*, almost all of which are clock-regulated. However, after adjusting for this phase difference, only a handful show overall expression level differences between *rve8-1* and wild type. Thus the apparent differential expression is mainly due to the phase difference between these two genotypes. These findings prompted us to examine the effect of phase on gene expression within a single genotype. Using samples of wild-type plants harvested at thirty-minute intervals, we demonstrated that even this small difference in circadian phase significantly influences the results of differential expression analysis. Our study demonstrates the robust influence of the circadian clock on the transcriptome and provides a cautionary note for all biologists performing genome-level expression analysis.

## Introduction

Circadian rhythms are physiological and behavioral cycles with period length around 24 hours that are produced by an endogenous clock [1]. They are widely observed in nature, presumably because they help organisms prepare for predictable environmental change such as day/night cycles. Circadian rhythms can be further classified according to their daily phases, or times of peak expression [2]–[5]. Different groups of circadian rhythms are timed to occur at specific times of day to optimize physiology and growth. Functional circadian clocks provide a crucial fitness advantage in diverse organisms: disruption of clock components results in disorder of sleep/wake cycles in humans [6] and reduction of photosynthesis and overall survival in cyanobacteria and plants [7], [8].

Clock systems can be generalized as consisting of three major parts [1]: (1) input pathways, which sense environmental timing cues such as light or temperature and can reset the clock, (2) the central oscillator (or the central clock), which consists of interlocking transcriptional/translational feedback loops, and (3) outputs, biological rhythms with a free-running period of approximately 24 hours. Although the identities of clock genes vary between different organisms, many clock components are transcription factors involved in regulatory feedback loops. In Arabidopsis, the first-identified transcriptional loop of the central clock is composed of three transcription factors: CCA1 and LHY, two MYB-like transcription factors highly expressed in the morning [9], [10], and TOC1, a CCT-domain containing transcription factor with peak abundance in the evening [11], [12]. The two morning-phased transcription factors repress *TOC1* expression by directly binding to a motif found in the *TOC1* promoter called the evening element (EE) [12], [13]. The EE is overrepresented in evening-phased genes and when multimerized confers evening-phased expression on a reporter gene [4], [13]. TOC1 was recently reported to be a transcription factor that directly inhibits *CCA1* and *LHY* expression [14], [15], revealing that these three proteins function in a double-negative feedback loop.

In plants as in other eukaryotes, multiple transcriptional feedback loops are coupled together to generate the circadian oscillator. A second negative feedback loop in Arabidopsis is formed between CCA1/LHY and three TOC1 homologs: PRR5, PRR7 and PRR9 [16]. We recently described another transcriptional loop involving one of these pseudoresponse regulators and *RVE8*, a homolog of *CCA1* and *LHY*. RVE8 promotes expression of *PRR5*, possibly by binding to the EE found in the *PRR5* promoter, and *RVE8* expression is in turn repressed by PRR5 [17].

Transcriptional regulation is not only key to oscillator function in all eukaryotes studied, but also plays an important role in control of clock outputs. In the past decade, microarrays have been widely used in many model systems to simultaneously monitor levels of thousands of transcripts in the genome. These studies have revealed that 9% to 30% of the transcriptome in cyanobacteria, Arabidopsis, Drosophila and mammals is controlled by the clock [2], [18]–[21]. Identification of pathways enriched for circadian-regulated genes has demonstrated that numerous essential metabolic and physiological pathways are influenced by the clock [2], [21], [22]. The examination of global transcript abundance over circadian time thus allows the identification of both components of the circadian oscillator as well as genes and pathways under circadian regulation.

Here we compare gene expression in the long-period Arabidopsis mutant, *rve8-1* [17] and in wild-type plants after several days of growth in constant environmental conditions. This prolonged time in free-running conditions resulted in a global 4-hour delay, approximately 17% of a daily cycle, in the phase of both central clock and output genes in the *rve8-1* mutant relative to wild type, demonstrating that RVE8 acts within the central clock. If we did not take this phase difference into account, a great number of genes were identified as differentially expressed; however, only very few of them showed significant changes in expression levels and/or patterns of gene expression once we compensated for the phase difference. This profound influence of phase on global gene expression prompted us to examine the effects of smaller phase differences on gene expression. Using publicly available microarray data, we demonstrate that even a phase difference as small as 30 minutes (2% of a daily cycle) has a dramatic effect on expression levels of hundreds of genes, a finding with important ramifications for the design of all genome-wide studies.

## Results

### RVE8 Affects Global Circadian-regulated Transcription

**Figure 1:**
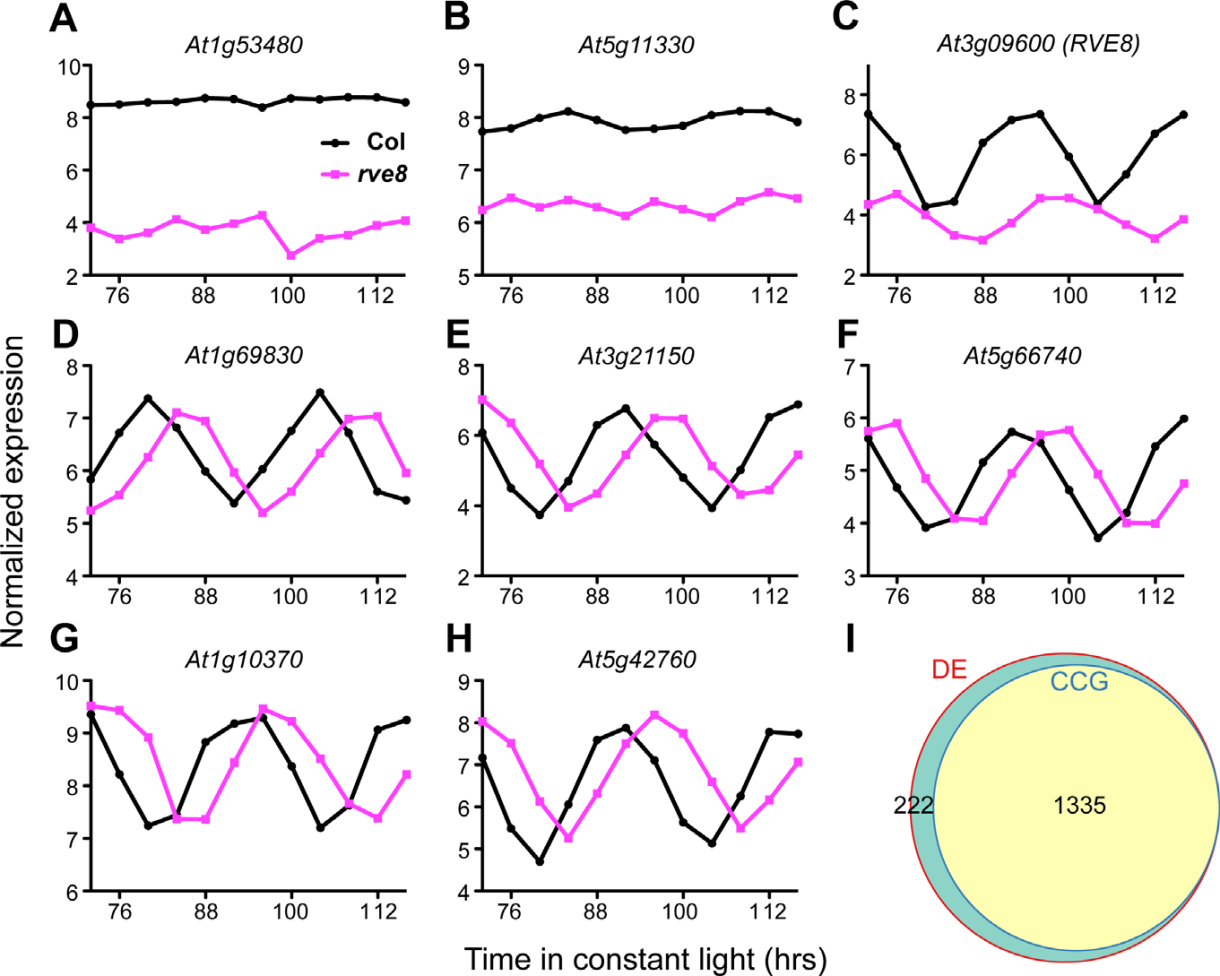
Most of the genes differentially expressed between Col and *rve8-1* are clock-regulated. Plants were entrained in light/dark cycles for 7 days before release to constant light and temperature (free run). Samples were harvested at 4-hour intervals over two days, starting after 72 hours in free run. RNA was extracted and labeled and then hybridized to tiling microarrays. (A–H) The expression patterns of the 8 most differentially expressed genes. The 72 and 96 hours in the x-axis correspond to subjective dawn. Genes expressed with a circadian rhythm (C–H) display a phase delay of about four hours in *rve8-1*, but only *At3g09600* (*RVE8*) also has an obvious expression level difference (C). (I) A weighted Venn diagram presents the relative portion of clock-controlled genes (CCGs) among differentially expressed (DE) genes.

**Figure 2:**
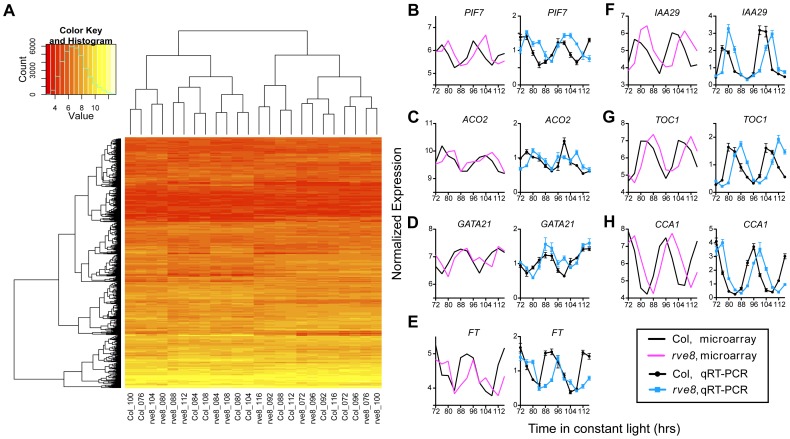
The circadian transcriptome of *rve8-1* has a 4-hour phase delay relative to wild type. (A) Hierarchical clustering of the 1557 genes differentially expressed between the Col and *rve8-1* time courses. Gene identifications are on the y-axis, time of sample collection and genotype are on the x-axis. The color key indicates the expression level in log_2_ scale (red to light yellow: low to high expression) and overall frequency of each expression level. (B–H) Validation of microarray results using qRT-PCR on clock-controlled genes with a range of amplitudes (shown from low to high amplitude). For each panel, the left part shows microarray data and the right shows qRT-PCR results (mean ± SEM from three technical replicates) for the gene examined.

To identify target genes controlled by RVE8, we examined the transcriptional profiles of Col and *rve8-1* using AGRONOMICS1 tiling arrays, which cover more than 30,000 annotated Arabidopsis genes [23]. Plants were grown in light/dark cycles for seven days and then transferred to free-running conditions (continuous light and temperature). Since *rve8-1* has a period only approximately one hour longer than wild type, we collected samples on the fourth and fifth days of free run to ensure easy detection of the circadian phase difference between Col and *rve8-1* [17]. Following preprocessing and normalization of raw array data, genes with very low expression levels were filtered out. An empirical Bayes statistical analysis using limma [24] was then performed to identify genes differentially expressed between the two genotypes, in which we matched the mutant and wild-type samples according to the time at which they were harvested (i.e. comparing Col_72 and *rve8-1*_72). Using a multiple-measure adjusted p value less than 0.05 as a cut-off, 1557 genes were identified as significantly differently expressed between Col and *rve8-1*. Further analysis of the time course data (see below) revealed that transcript levels of 86% of these misexpressed genes are controlled by the circadian clock (Fig. 1I). These differentially expressed genes include key clock genes such as *TOC1* and *CCA1* (Fig. 2G–H) as well as a variety of output genes (Fig. 2B–2F). Alteration of both central clock and output genes in *rve8-1* is consistent with previous reports that suggest RVE8 functions close to the central clock in Arabidopsis [17], [25] and thus would be expected to directly or indirectly regulate all clock-controlled genes (CCGs). Inspection of the most highly differentially expressed genes that are also clock-controlled show that they all have a delayed phase in *rve8-1* compared to Col, but reveal no obvious change in overall expression levels (Fig. 1C–1H), with the exception of *RVE8* (*At3g09600*) itself (Fig. 1C).

Hierarchical clustering of the 1557 differentially expressed genes showed a strong circadian signature. That is, the Col samples harvested after 76 and 100 hours in free run (24 hours apart) cluster with each other, as do the *rve8-1* samples harvested after 80 and 104 hours in free run (Fig 2A). Notably, these four time points also cluster with each other. A similar relationship was seen between all the other time points; i.e., the Col samples cluster most tightly with the *rve8-1* samples collected four hours later. Validation of microarray results using qRT-PCR on CCGs that cycle with both low and high amplitudes showed good agreement between the two techniques (Fig. 2B–H), indicating the microarray results are reliable. These data show that after four days in free-run, global circadian gene expression in *rve8-1* is delayed about four hours when compared to Col. The four-hour phase difference after four days in free run can be attributed to the approximately one hour longer period in *rve8-1* mutants [17], [25]. The lack of apparent overall expression level differences in most of the “differentially expressed” genes once phase is considered (Fig. 1D–I and Fig. 2B–H) and the close clustering of the wild-type and *rve8-1* samples (Fig. 2A) imply that the majority of the 1557 genes identified as differentially expressed simply have a different circadian phase in *rve8-1*, rather than an overall change in expression levels.

### Identification of RVE8 Targets with Altered Expression Levels

**Figure 3:**
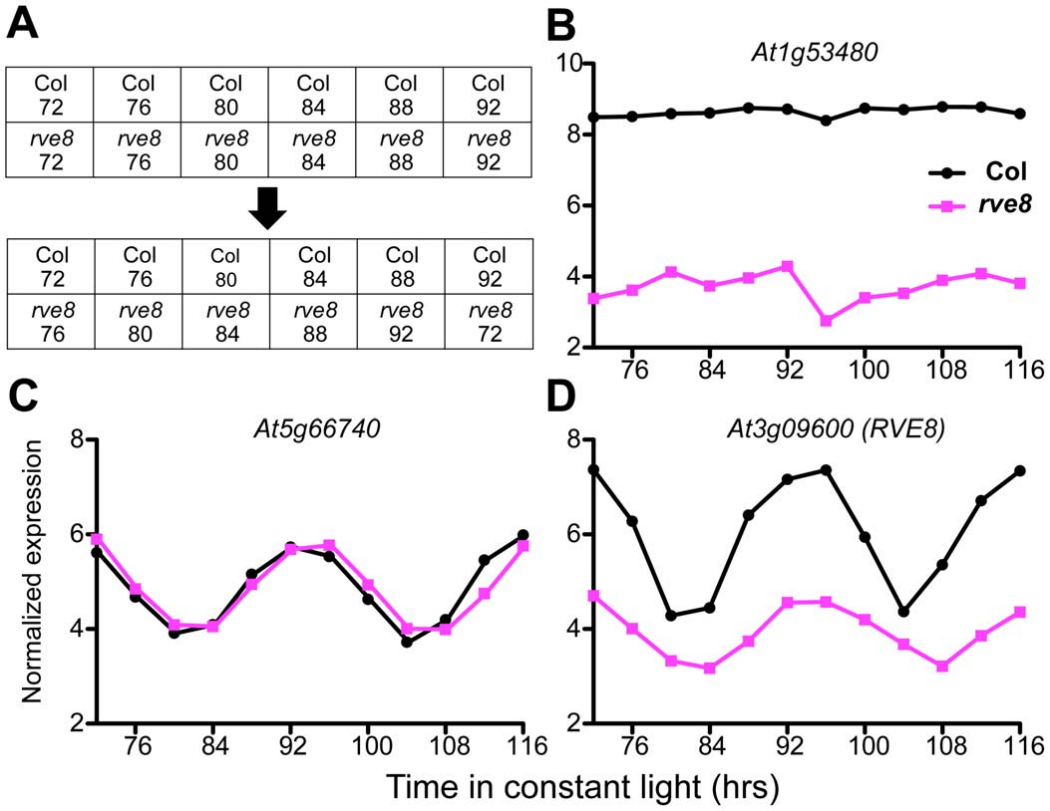
Comparison of transcriptional profiles after compensation for the phase difference. (A) Diagram shows the time points compared to each other using limma either without phase adjustment (upper panel) or with phase adjustment (lower panel). After compensating for the phase difference, Col_72 is compared to *rve8*_76, and so on. (B–D) New time course data alignments of genes previously classified as the most significantly differentially expressed after adjusting for the phase difference. The time shown on the x-axes is that of the Col samples.

**Figure 4:**
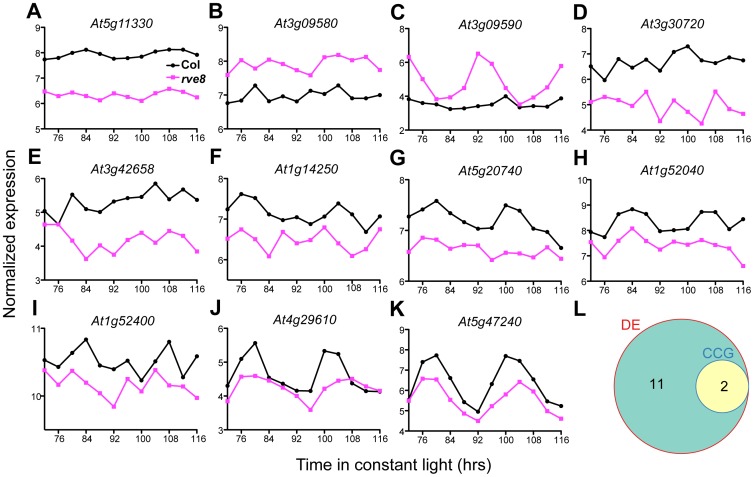
Genes differentially expressed between Col and *rve8-1* after phase adjustment. (A-K) Genes with overall differences in expression levels in *rve8-1*, graphed with the phase adjustment described in the main text. The time shown on the x-axes is that of the Col samples (72 and 96 hours corresponds to subjective dawn). These 11 genes (along with *At1g53480* and *RVE8* shown in Fig. 3B and 3D) are the only ones identified as misexpressed in *rve8-1*. (L) Two out of the 13 differential expressed genes (DE) are classified as clock-controlled genes (CCGs) in both Col and *rve8-1* (*At4g29610* in Fig. 4J, and *At5g47240* in Fig. 4K). Note that *At5g20740*, *At1g52040*, and *At3g09600* (*RVE8*, Fig. 3D) which cycle in Col but not in *rve8-1* (G, H), are not classified as CCGs in Fig. 4L.

Since we wished to identify genes with overall alterations in expression levels in *rve8-1*, not just those with altered phases, we adjusted the two time series to compensate for the observed four-hour difference in phase (Fig. 1 and 2) and then carried out differential expression analysis. That is, we used limma as described previously, but this time compared Col_72 with *rve8-1*_76, Col_76 with *rve8-1*_80, etc. (Fig. 3A) We expected four possible types of outcomes for genes previously identified as differentially expressed: (1) genes controlled by the clock that do not have appreciable differences in overall expression levels in *rve8-1* would no longer be identified as differentially expressed, (compare Fig. 1F and Fig. 3C); (2) differentially expressed genes that are not controlled by the circadian clock would still show expression level differences (compare Fig. 1A and Fig. 3B); (3) genes controlled by the clock that also have changes in expression levels (like *RVE8*, compare Fig. 1C and 3D) or a change in phase other than a four hours delay in *rve8-1* would be still recognized as differentially expressed genes; and (4) genes that are clock-regulated in only one of the genotypes (Fig. 4C, 4G, and 4H) might also be differentially expressed. After comparison of these “offset” Col and *rve8-1* time courses, only 13 genes passed the significance criteria for differential expression (adjusted p value < 0.05; Fig. 3B, 3D, and Fig. 4). Given that most of the 1557 genes initially identified as differentially expressed are also circadian regulated (Fig. 1I), the vast majority of these genes fall into our class 1, simply having a phase delay in *rve8-1* rather than an overall expression level difference between wild type and the mutant.

The 13 genes that are differentially expressed in the phase-offset datasets fall into each of the above classes 2 – 4. Two of the 13 (*At4g29610*, and *At5g47240*) are clock-regulated in both genotypes (4J–L). The two genes upstream of the *RVE8* locus, *At3g09580* and *At3g09590* (Fig. 4B and 4C), are also misregulated in *rve8-1*, suggesting the T-DNA insertion in *RVE8* not only disrupts *RVE8* expression but also affects nearby genes. Interestingly, expression of *At3g09590* is clock-regulated with a dawn phase in *rve8-1* but not in Col, suggesting that the enhancer elements within the T-DNA inserted in the *RVE8* locus have imposed *RVE8*-like expression patterns on this adjacent gene. Four evening-phased CCGs (*At5g20740*, *At1g52040*, *At4g29610*, and *At5g47240*) have reduced expression levels in *rve8-1*; two of them lost rhythmic expression in *rve8-1* (Fig. 4G and 4H) and two are still classified as clock-regulated despite reduced levels in *rve8-1* (Fig. 4J and 4K). None of these 13 differentially expressed genes (except *RVE8* itself) have been reported to affect the circadian clock and are annotated with diverse functions in plants (Table 1). Our results show that after correcting for circadian phase only a handful of genes have significantly altered expression levels in *rve8-1.*

**Table 1:** Annotations for the genes with differential expression levels in *rve8-1* after correction for circadian phase.

AGI	Short description
At1g53480	unknown protein
At5g11330	monooxygenase family protein
At3g09600	myb family transcription factor (RVE8)
At3g09580	amine oxidase family protein
At3g09590	pathogenesis-related protein, putative
At3g30720	QQS (QUA-QUINE STARCH)
At3g42658	transposable element gene
At1g14250	nucleoside phosphatase family protein / GDA1/CD39 family protein
At5g20740	invertase/pectin methylesterase inhibitor family protein
At1g52040	MBP1 (MYROSINASE-BINDING PROTEIN 1); protein binding
At1g52400	BGLU18 (BETA GLUCOSIDASE 18); catalytic/ cation binding / hydrolase, hydrolyzing O-glycosyl compounds
At4g29610	cytidine deaminase, putative / cytidine aminohydrolase, putative
At5g47240	atnudt8 (Arabidopsis thaliana Nudix hydrolase homolog 8); hydrolase

### Investigation of Rhythmic Expression and Generation of a More Complete Clock-regulated Gene List

**Figure 5:**
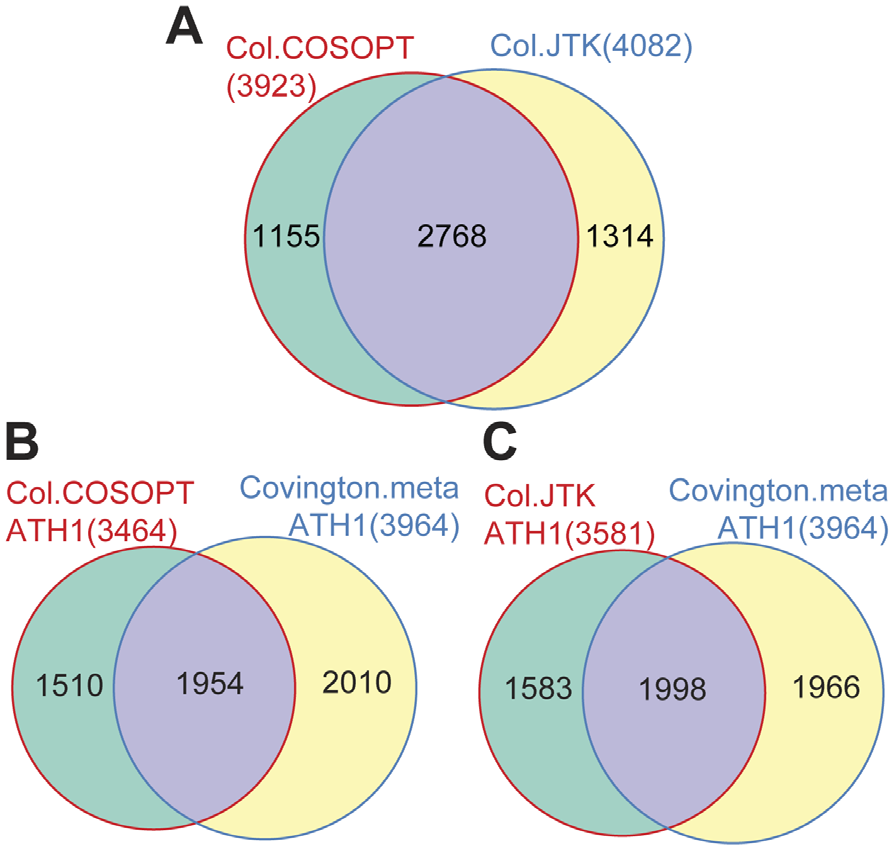
Comparison between lists of clock-regulated genes identified using separate algorithms. (A) A weighted Venn diagram comparing clock-controlled genes (CCGs) identified using COSOPT and JTK_CYCLE from our Col data. (B-C) Comparing the CCG lists generated from our Col data using either COSOPT or JTK_CYCLE, including only genes also represented on the ATH1 array, with the list of CCGs generated by Covington et al. [2]. Covington’s CCG list was generated using COSOPT to analyze a meta time course dataset, which combined two independent circadian time course experiments performed using the ATH1 microarray platform.

In addition to expression levels, we also examined circadian waveforms in Col and *rve8-1*. Given that only a dozen genes have different expression levels between the two genotypes once phase is taken into account, we expected little or no difference in rhythmic pattern change except the phase delay in *rve8-1*. We first compared the performance of a commonly used method, COSOPT [26], and a relatively new algorithm, JTK_CYCLE [27], for detecting cycling genes in Col. Both algorithms test correlations between the experimental time course data and a series of cosine models [26], [27], however, JTK_CYCLE uses a nonparametric test and was reported to identify rhythmic genes more reliably with enhanced resistance to outliers and improved computational efficiency [27]. Based on previous studies [2], [4], [27], [28] and our empirical tests, a pMMCβ < 0.05 for COSOPT and multiple-measure adjusted p vlaue < 0.05 for JTK_CYCLE were chosen as significance thresholds. JTK_CYCLE identified slightly more cycling genes than COSOPT in Col (4082 found by JTK_CYCLE compared to 3923 found by COSOPT) with 68 to 71% of the genes shared between the two lists (Fig. 5A). We also examined the agreement between these gene lists and that obtained in a previous study by Covington et al. performed using ATH1 arrays [2]. This previous list was produced using COSOPT to analyze a meta-dataset generated by combining two independent time courses, both from Col plants [2]. Considering only genes also represented on the ATH1 array, our current CCG lists produced by JTK_CYCLE and COSOPT have similar overlaps with the Covington gene list, with slightly better coverage provided by the JTK_CYCLE list (Fig. 5B – C). The agreement between either our COSOPT or our JTK_CYCLE list with Covington’s list (∼50%) is greater than a previous report showing around one-third of CCGs overlapping between independent circadian microarray studies [2], suggesting our CCG lists generated using Agronomics1 arrays are reliable. Since JTK_CYCLE takes only about 1/32 of the computing time required by COSOPT and the performance of the two algorithms is similar (Figure 5), we believe JTK_CYCLE to be better suited for analysis of large data sets.

To compare rhythmic gene expression in Col and *rve8-1*, we determined the cycling genes lists in *rve8-1* by JTK_CYCLE using the same criteria as described above. A similar number of cycling genes are found in *rve8-1* and in Col (3635 and 4082, respectively). Furthermore the overlap between the CCGs found in Col and in *rve8-1* is about 44 and 49% of each data set (Fig. S1), which is similar to what we observed when comparing our Col data set with a previously published list generated using Col plants (Fig. 5B–C). These results suggest circadian expression patterns are not strongly different between Col and *rve8-1*, consistent with the very small number of genes we find differentially expressed between these genotypes after we have compensated for phase (Fig. 4). Inspection of the genes only classified as clock-regulated in one genotype suggest that these genes are actually clock-regulated in the other genotype as well. For example, *FT* is classified as cycling in Col but not in *rve8-1* by JTK_CYCLE, but the transcript shows reasonable cycling in both genotypes by both microarray and qRT-PCR analysis (Fig. 2E). This suggests that circadian analysis of single genotypes, with only 12 time points considered per time course, results in many CCGs being missed due to insufficient statistical power.

**Figure 6:**
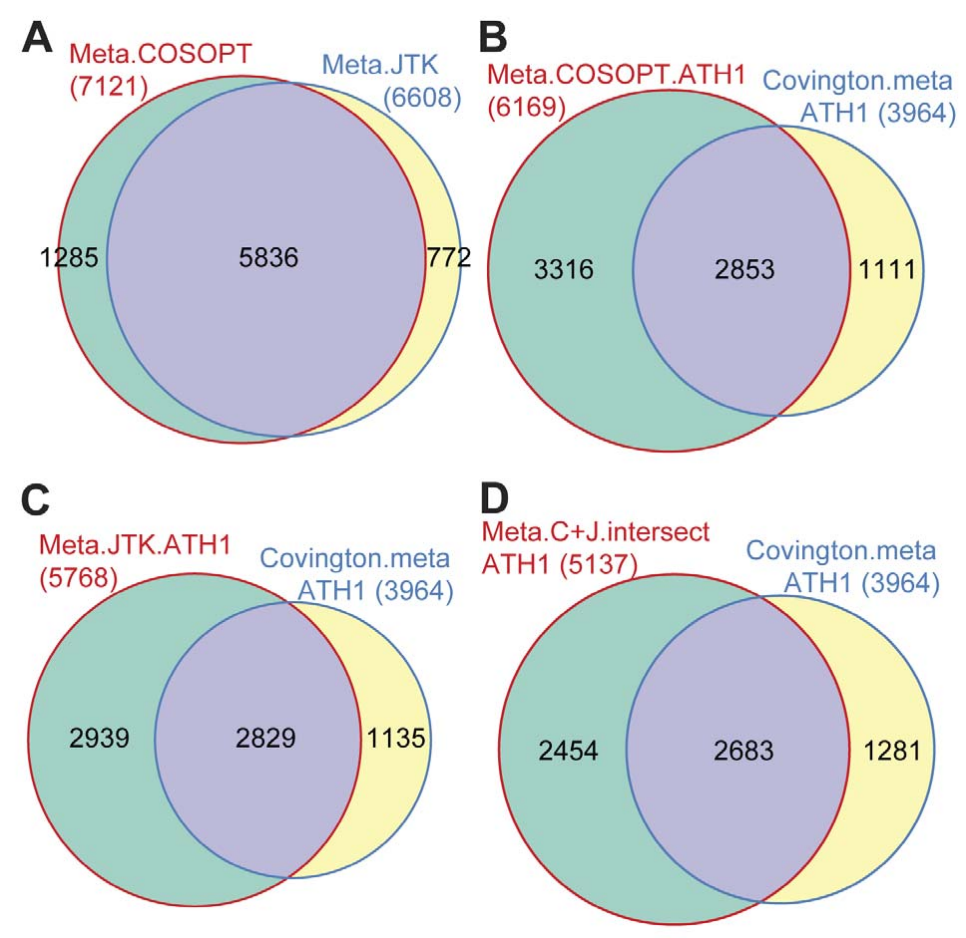
Comparison of circadian-regulated gene lists generated using meta time course data. Col and *rve8-1* time courses were combined (in the order Col_76…Col_116, *rve8*_76, *rve8*_80, …, *rve8*_116, *rve8*_72) to create a meta time course data set spanning four days. Clock-controlled genes (CCGs) were identified either using COSOPT or JTK_CYCLE. (A) A weighted Venn diagram comparing CCGs in the meta time course data identified using either COSOPT or JTK_CYCLE. (B) Comparison of CCGs identified using COSOPT to analyze the combined Col and *rve8-1* meta data set with CCGs identified in a previous study done using ATH1 arrays [2]; only genes also represented on ATH1 arrays were considered. (C) Comparison of CCGs identified using JTK_CYCLE to analyze the combined Col and *rve8-1* meta data set with CCGs identified in a previous study done on ATH1 arrays [2]; only genes also represented on ATH1 arrays were considered. (D) Comparison of CCGs identified by both COSOPT and JTK_CYCLE with CCGs identified in a previous study done on ATH1 arrays [2]; only genes also represented on ATH1 arrays were considered.

Covington et al have reported a solution to improve the low statistical power common in many circadian microarray studies: combining data sets from different time courses with similar growth conditons to create a meta-time course allowing more reliable and robust detection of circadian rhythms [2]. Since there is little appreciable difference in the transcriptional profiles between Col and *rve8-1* once phase is accounted for (Fig. 2A), we combined these two time courses to create a four-day meta-time course consisting of 24 separate samples. Upon analysis of this four-day meta-time course with COSOPT and JTK_CYCLE, we identified 7021 (Table S1) and 6608 genes (Table S2) as clock-controlled, respectively (Table 2). Notably 5836 genes overlap between the two CCG lists (corresponding to 83% and 88% of genes identified by COSOPT and JTK_CYCLE, respectively, Fig. 6A). We next compared the lists of cycling genes generated using our 24 sample (Col + *rve8-1*) meta time course with that generated by Covington et al. using a 25 sample (Col + Col) meta time course [2]. Restricting our analysis to genes represented on the ATH1 arrays, we found that the CCG lists generated by either COSOPT or JTK_CYCLE cover about 71–72% of the genes defined as CCGs by Covington using COSOPT (Fig. 6B–C). This is much better agreement than we found using only our Col time course (Fig. 5B–C), suggesting that combining the Col and *rve8-1* time courses to generate one meta data set enhanced identification of CCGs.

**Table 2:** Summary of the Agronomics1 array data generated in this study.

	Transcripts represented onAgronomics1 array	Transcripts also represented on ATH1 array
Transcript number	30237	22591
Expressed transcripts	19961	16341
CCGs identified by COSOPT^1^	7021	6169
CCGs identified by JTK^1^	6608	5840
CCGs identified by both COSOPT and JTK_CYCLE^1^	5836	5137
CCG percentage (%)^2^	29.2	31.4

1 CCGs identified using the meta time course data.

2 CCG percentage based on the genes identified by both COSOPT and JTK_CYCLE.

We next compared the CCGs identified by both COSOPT and JTK_CYCLE with those only identified by either one or the other method. Genes found to be clock-regulated only by COSOPT or only by JTK_CYCLE have significantly lower amplitudes than those identified by both methods (Fig. S2). This suggests that genes only identified by one method are less robust cyclers than those identified by both methods. We therefore considered the list of genes identified as CCGs by both COSOPT and JTK_CYCLE as CCGs for subsequent analyses; although we are likely omitting true clock-regulated genes, we have high confidence in the genes included in this group. Indeed this gene set, representing the intersection between the COSOPT and JTK_CYCLE lists, still includes ∼68% of Covington’s CCGs (Fig. 6D). The 1281 genes excluded from our CCG list but present in Covington’s list (Fig. 6D) might include genes whose rhythmic expression damps after a few days in free-run, as Convington’s meta set combines two circadian time courses collected on days 2 and 3 of free run while our samples were harvested on days 4 and 5 of free-run. Our meta list created using the Agronomics1 array includes 3133 genes not previously reported as CCGs by Covington et al. (Fig. S3), of which 699 genes are not represented on the ATH1 array. These additional CCGs include many well-documented clock-regulated genes, such as *PRR5*, *GI*, *LUX* and *CO* (Table S1 and S2). Overall, our cycling list shows good agreement with the previously identified CCG list, with a similar fraction of the transcriptome estimated to be clock-regulated in the two experiments [2] (Table 2). We also validated some newly identified CCGs with either low or high amplitudes using qRT-PCR (Fig. 2B–2F). The reproducible detection of rhythmic gene expression using both microarray and qRT-PCR techniques further suggests our CCG list generated by analysis of the combined data sets is reliable. This more complete list of CCGs may enable new insights into plant physiology.

### Phase Differences as Small as Half an Hour can Result in Significantly Different Levels of Gene Expression

**Figure 7:**
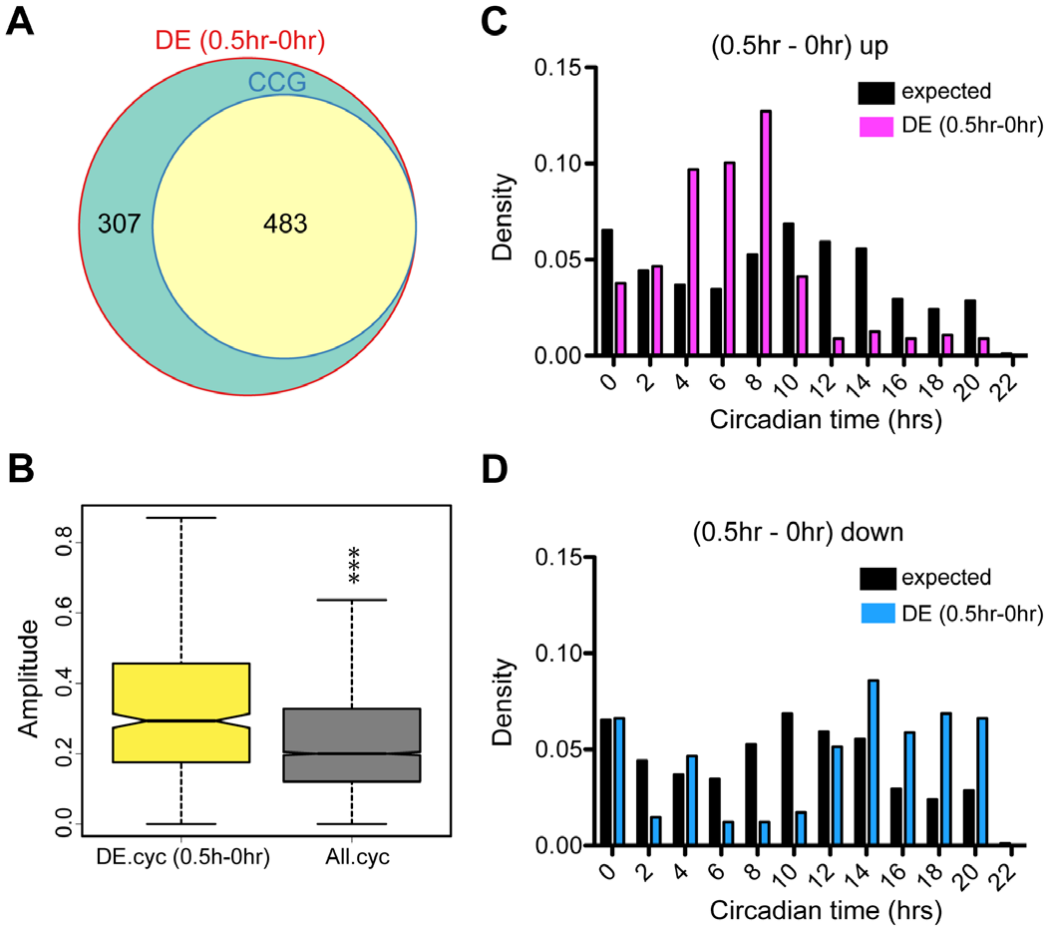
Small differences in circadian phase have profound effects on differential expression analysis. Differential expression analysis using a publicly available dataset of Col harvested at time 0 (corresponding to circadian time 3, i.e., 3 hours after dawn, herein defined as “0 hr”) and 30 minutes later (herein defined as “0.5 hr”) [29]. (A) A weighted Venn diagram presenting the proportion of clock-controlled genes (CCGs) among genes found to be differentially expressed between the 0 and 0.5 hour samples (DE[0.5 hr-0 hr]). (B) A box plot comparing the circadian amplitudes of the differentially expressed CCGs (DE.cyc[0.5 h-0 hr]) and those of all CCGs. The differentially expressed CCGs have on average significantly higher circadian amplitudes (p < 2.2e-16, Wilcox test). (C–D) The phase distributions of the CCGs classified as “up-regulated” or “down-regulated” between 0.5 and 0 hours are plotted alongside the observed phase distribution of all identified CCGs (expected).

The phase difference between Col and *rve8-1* after four days in free-run is around four hours [17], which had a strong effect on differential expression analysis despite limited changes in overall gene expression levels (Figs. 1 and 4). To further investigate potential effects of phase on determination of differential expression, we used publicly available microarray datasets to compare gene expression in Col vegetative shoots harvested at 30-minute intervals [29]. In this experiment, plants were grown in light/dark cycles and time 0 was defined as three hours after lights on, which corresponds to circadian time 3 (CT3). We examined gene expression levels in Col sampled at times 0, 0.5 and 1 hours. Comparing the 0 and 0.5 hour (CT3 and CT3.5) samples, 790 genes are differentially expressed according to our criteria (adjusted p value < 0.05, with analysis carried out using limma). Of these, 483 are classified as clock-controlled in our meta data set described above (∼61%), much more than the ∼1/3 expected by chance (Fig. 7A and Table 3). We also found a greater fraction of CCGs than expected when we compared the 0.5 hour and 1 hour (CT3.5 and CT4) time points (∼50% of the 1859 differentially-expressed genes are clock regulated) (Fig. S4A and Table 3). In both gene lists, CCGs are significantly overrepresented (p < 2.2 e-16; Table 3).

**Table 3:** Summary of differential expression analysis in the Col short sampling interval datasets.

Comparison	DE^1^	CCGs^2^	CCG%^3^	Median amplitude^4^
0.5 hr - 0 hr	790	483 ***	61.1	0.2934 ***
1 hr - 0.5 hr	1859	930 ***	50.0	0.2482 ***

1 Differentially expressed in the indicated comparison and passing the expression filter in our Agronomics1 tiling array experiment;

2 clock-controlled genes identified in the meta time course set by both COSOPT and JTK_CYCLE;

3 the percentage of CCG in DE list;

4 Amplitudes were determined using JTK_CYCLE.

Clock-controlled genes (CCGs) are overrepresented among the genes differentially expressed in samples harvested at 30 minute intervals (Fisher’s exact test on both sets, p < 2.2e-16, ***) when compared to the approximately 1/3 of CCGs in the genome. These differentially expressed cycling genes also have significantly higher circadian amplitudes (Wilcox’s test on both sets, p < 2.2e-16, ***) than all CCGs in the genome (median amplitude for all CCGs = 0.2001).

If these CCGs are classified as differentially expressed solely due to a phase difference between the times the plants were harvested, we would predict that the up-regulated genes would be enriched for a certain range of phases while the down-regulated genes would be enriched for the opposite/complementary phases. In accordance with this prediction, genes with peak circadian expression 4 to 8 hours after dawn (CT4 – CT8) are overrepresented and oppositely phased genes are underrepresented amongst the genes that are up-regulated in our “0.5 vs 0 hour” comparison (Fig. 7C). Thus clock-regulated genes with peak expression during the day (CT4–CT8) have higher expression levels in the samples collected slightly later in the day (0.5 hr, or CT3.5) than those collected at 0 hr (CT3), because these genes are approaching their peak levels. Also as predicted, the phase distribution of the down-regulated genes shows the opposite pattern as the up-regulated genes: the down-regulated genes are enriched for those with peak expression between CT14–20 and deficient for genes with peak expression between CT2–10 (Fig. 7D). Thus clock-regulated genes with peak expression during the night have higher expression levels in the samples collected earlier in the day (0 hr, or CT3), consistent with the differential expression being due to a phase difference. Similar patterns were observed when comparing the 1 and 0.5 hour time points, but with slightly shifted enriched phase distributions (Fig. S4C–D). This slight shift in the enriched phase is expected as genes peaking at times close to the new time points being examined will be affected more strongly, indicating time of sampling is also critical for phase effect. Also consistent with the differential expression of CCGs being due to a phase difference, these differentially expressed genes have on average higher amplitude circadian expression than do all CCGs in the genome (Fig. 7B and Fig. S4B). In conclusion, these results demonstrate that even very small differences in circadian phase (30 minutes in this example) can cause significant differences in expression in large numbers of genes, especially those with high circadian amplitudes.

## Discussion

### The Role of RVE8 in the Central Clock in Arabidopsis

Previous studies have shown that RVE8 affects the phase of both central clock genes (*CCA1*, *LHY*, and *TOC1*) and output genes (*CCR2*) in both loss-of-function T-DNA mutants and in overexpression lines [17], [25]. Since the light input pathway was not appreciably altered in these genotypes [17], these data suggested RVE8 functions close to the central oscillator in Arabidopsis. By determining the transcriptional profiles of Col and *rve8-1* in the current study, we have found that as expected given the one hour long period phenotype of *rve8* mutants, all of the central clock components and output genes display an approximately four-hour delay in *rve8-1* after four days in free-run (Fig. 1 and Fig. 2). This further supports the hypothesis that RVE8 plays a role in the central clock in Arabidopsis.

After adjusting for the phase difference between these two time courses, we identified very few genes with altered expression levels or patterns in the *rve8-1* mutant (Fig. 3 and Fig. 4). Among the 13 genes that do show defects in gene expression not accounted for by phase, we identified four evening-phased CCGs (*At5g20740*, *At1g52040*, *At4g29610*, and *At5g47240*) (Fig. 4G–H and 4J–K). All four genes have reduced expression levels in *rve8-1*, while two of them also lose the rhythmic pattern. RVE8 has been shown to bind the EE *in vitro* and *in vivo*, and has peak protein levels in the subjective afternoon [17]. Intriguingly, one or more EE-like sequences (AAATATCT or AAAAATCT) are found within 1500 bp upstream of the transcriptional start sites of all four of these genes (Figure S5), suggesting they might be direct targets of RVE8. The reduced levels of these four evening-phased genes in *rve8-1* supports the idea that RVE8 promotes evening gene expression, perhaps via modulation of histone 3 (H3) acetylation [25].

Given the clear circadian phenotype in *rve8-1* after four days in free-run, it was surprising that only a few genes show overall changes in expression levels. One explanation for this might be partial genetic redundancy within the RVE/CCA1/LHY gene family. There are 11 members in this family in Arabidopsis, each containing a signature single MYB-like domain followed by a proline-rich region. The closest homologs to RVE8 are RVE3, RVE4, RVE5, and RVE6, of which all but RVE3 have also been reported to associate with EE motifs [17]. Another possible explanation could be that RVE8 acts in the afternoon to promote evening-phased gene expression, affecting the timing of onset of transcript accumulation, but does not appreciably influence peak or trough transcript levels. According to this model, the *rve8-1* long period phenotype might be due to a delay in accumulation of evening-phased clock genes rather than to overall changes in their expression levels. Important future directions will be to examine the expression levels of evening genes in plants mutant for multiple *RVE* genes and to investigate the role of RVE8 on the precise timing of clock gene expression.

### Identification of Circadian-regulated Genes

A number of time course microarray experiments on Arabidopsis have been conducted to identify circadian-regulated genes [3], [4], [22], [30], [31]. However, only a low percentage of genes (32–37%) were found to overlap between clock-regulated gene lists generated using separate datasets [2]. This could be due to insufficient statistical power given the low sampling resolution (four hour intervals), small number of replicates, and short time courses (two days) in these experiments [2], [28]. By combining two independent microarray datasets followed by detection of rhythmic expression, Covington et al. found a higher number of cycling genes (3975 genes) that captured 79–87% of the clock-regulated transcripts identified in individual datasets [2]. These results demonstrate that the use of meta-data allows the more reliable identification of cycling genes and significantly increases the agreement between different data sets.

Given the close similarity between our wild-type and *rve8-1* datasets, we used the approach previously described by Covington et al. [2] and integrated these two independent datasets before identification of CCGs. We found that 6608 out of 19961 expressed genes (33%) are classified as CCGs using JTK_CYCLE and a similar number of cycling genes (7021, 35%) are identified using a second method, COSOPT. This ratio of cycling genes is similar to the 36% of the expressed genes identified as CCGs by Covington et al. [2]. Notably, 5835 genes were found in common between the CCGs identified by either COSOPT or JTK_CYCLE. Furthermore, when we restricted our CCG list to the intersection of CCGs identified by both methods, our gene list still contains 68% of the genes identified in the Covington’s study. This is remarkably good agreement considering this previous study used a different microarray platform and identified CCGs using only COSOPT. Indeed, the agreement between our CCG list and that of Covington et al. is greater than that previously found when comparing independent circadian microarray studies [2].

In addition to reliably identifying genes previously designated as CCGs, we also identified 3133 CCGs not included in the Covington et al. list. 699 of these are not represented on ATH1 arrays; the remaining 2434 may have been identified as CCGs in our but not previous experiments because Agronomics1 tiling arrays are reported to yield more reliable expression levels than ATH1 arrays [23]. In contrast, Covington’s list contains 1135 CCGs that are not identified in our data sets. This may in part be due to differences in sampling times, as Covington’s list was generated from two time courses collected on the 2^nd^ and 3^rd^ day in free-run while our samples were harvested on the 4^th^ and 5^th^ day in free-run, at which point some less robust circadian rhythms may have damped. Despite this, our analysis has generated a more complete cycling list that includes a number of previously missed, well-documented, clock-regulated genes as well as novel clock-regulated genes. This more complete gene list will provide a useful reference for future study, especially for analysis of genes not represented on the ATH1 array.

### Even Small Differences in Circadian Phase have Strong Effects on Gene Expression

We have demonstrated that without taking phase into account, 1557 genes are identified as differentially expressed between Col and *rve8-1*, although follow-up analysis revealed that very few genes showed alterations in overall expression levels or patterns between these genotypes. Remarkably, we also found that even a 30-minute difference in phase (just 2.1% of a 24 hour period) results in the statistically significant misexpression of hundreds of genes. Most of these differentially expressed genes (50–61%) are CCGs (Fig. 7A and S4A, and Table 3), underscoring the importance of the circadian clock in genome-wide regulation of transcription. However, the effect of circadian phase on the observed differential expression could still be underestimated. Hughes et al. compared the numbers of CCGs identified using samples collected at 1-, 2-, 3-, or 4-hour intervals over two days [28]. They found that many fewer clock-regulated transcripts were identified using samples collected at 4-hour intervals, as done in our and most other circadian studies, than in the other sampling regimes. In addition, genes with ultradian rhythms (periods of 8 or 12 hours) could only be detected when samples were collected at intervals of less than 4 hours [28]. Therefore even more of the genes identified as differentially expressed in these samples may be clock-regulated than we estimate. Furthermore, other biological periodic rhythms may contribute to phase differences between samples. For example, 416 genes (out of 6220 genes in the genome) in *Saccharomyces cerevisiae* and approximately 700 genes in human cells grown in tissue culture have been reported to show cell-cycle-associated rhythms [32], [33].

Our study provides a cautionary note for all biologists: one should meticulously take the “time” into account when designing experiments, always harvesting control samples at the same time (more precisely, at the same phase) since differences as small as 30 minutes in time of sampling can have profound effects on experimental results. For large experiments that require a lengthy sample collection times, randomization of genotypes and replicates will help minimize the profound effects of phase on expression levels. We further suggest that investigators determine if CCGs are overrepresented amongst their differentially expressed genes; if so, this might be a sign that the differential expression is at least partially due to phase differences between the samples. Finally, while our current study has focused on transcriptional regulation, the circadian phase difference that we observed would likely also affect other physiological processes such as metabolic events, suggesting it is important to take time of day into account when designing all types of large-scale experiments.

## Materials and Methods

### Plant Material, RNA Processing and Array Hybridization

Seeds of *rve8-1* [17] and Col-0 (originally obtained from Lehle Seeds) were sterilized, plated on Murashige and Skoog (MS) agar media containing 3% sucrose, and stratified at 4°C in the dark for two days. Seedlings were then grown under 12 hours light (50–60 µmol m^−2^s^−1^ white fluorescent light):12 hours dark conditions at 22°C for 7 days before being released into constant white light. Samples (∼50 plants per samples) were collected every four hours on the 4^th^ and 5^th^ days in free-run, immediately frozen in liquid nitrogen, and then stored at –80°C until being processed. Total RNA was prepared with TRIzol reagent (Invitrogen), treated with RNase-free DNase I (Qiagen), and further purified using the RNeasy MinElute Cleanup Kit (Qiagen). The quality of the isolated RNA was determined by NanoDrop ND 1000 (NanoDrop Technologies) and Bioanalyzer 2100 (Agilent). Samples with a 260 nm: 280 nm ratio and a 260 nm: 230 nm ratio between 2 and 2.3, and an RIN (RNA integrity number) value greater than 8 were processed further. Biotin-labeled cRNA was prepared using the GeneChip 3′ IVT Express kit (Affymetrix) and hybridized onto Affymetrix AGRONOMICS1 Arabidopsis tiling arrays as previously described [23].

### Agronomics1 Array Data Analysis

Analysis was performed using an open source software under the Bioconductor [34] project with the statistical programming language R version 2.13 [35]. The poorly performing probes (since probes on the tiling array are restricted to fixed windows along the chromosome, the sequences of 25mer oligonucleotides may not have optimal hybridization properties) were dynamically masked during the analysis using the scripts provided by Rehrauer et al. [23] and were excluded from probe set summaries. Background correction, normalization, and summarization of probe sets were based on the Agronomics1 “all genes” CDF files with TAIR.9 annotation [23] and Robust Multichip Averaging (RMA) [36] implemented in the Aroma.Affymetrix package [37]. After this low-level analysis, genes with at least four time points showing expression levels greater than log_2_4.5 in either the Col or the *rve8-1* time course were considered to be expressed, and were used for the downstream analysis. Differential analysis was performed using the limma (Linear models for microarray data) package [24]: briefly, the 5^th^ day data were treated as replicates of the 4^th^ day data in each genotype. Accordingly, six time points with two replicates in Col and *rve8-1* were included in the linear model, and then empirical Bayes statistics were applied to determine the differential expression between the comparisons. An adjusted p value of less than 0.05 was used as a significance threshold. A heat map of the hierarchical clustering of the differentially expressed genes was created using the heatmap.2 function implemented in *gplots* package [38]. For comparing gene lists, weighted Venn diagrams were created using the *Vennerable* package [39]. For determining the significance of the relative proportions of clock-controlled genes in the differentially expressed gene lists in a given comparison and in the whole transcriptome, Fisher’s exact test was performed using the fisher.test function in R.

The raw data and normalized results have been deposited in the Gene Expression Omnibus database (GEO) (http://www.ncbi.nlm.nih.gov/geo) with accession number GSE37278.

### qRT-PCR

RNA was isolated using TRIzol (Invitrogen) and was treated with DNase I (Qiagen). cDNA was synthesized using SuperScriptase ΙΙ (Invitrogen) following the manufacturer’s protocol. qRT-PCR was performed as previously described [40]. Three technical triplicates for each sample were run using an iQ5 Real Time PCR machine (Bio-Rad), and starting quantity was estimated from critical thresholds using the standard curve method. Data were normalized to the respective *PROTEIN PHOSPHATASE 2A* (*PP2A*) expression levels in each sample. The primer sets for each transcript are listed in Table S3.

### Identification of Cycling Genes Using JTK_CYCLE and COSOPT

The Col and *rve8-1* two-day time course data were first analyzed separately using JTK_CYCLE to identify clock-controlled genes in each genotype. Genes with a period ranging from 20 to 28 hours and an adjusted p value less than 0.05 are considered cycling. In order to obtain more statistical power for the identification of clock-regulated genes, the Col and *rve8-1* time courses were combined (in the order Col CT_72, 76, …116, followed by *rve8*_76, 80, … 116, 72) to create a four-day meta time course, and circadian-regulated genes were identified using two independent cycling-detecting algorithms, JTK_CYCLE and COSOPT. In both cases, we required the estimated circadian period to be between 20–28 hours. Based on previous studies [26], [27] and our empirical tests, the significance thresholds for identification of clock-regulated genes were set to an adjusted p-value < 0.05 for JTK_CYCLE and a pMMC-β < 0.05 for COSOPT. For genes with estimated periods other than 24 hours, phase was adjusted according to the ratio of its period to 24 hours.

### Analysis of AtGenExpress Data

The raw data from AtGenExpress ME00325 [29] was normalized using RMA [36] before differential expression analysis was carried out using limma [24]. All time points (0, 0.5, 1, 3, 6, 12, and 24 hrs) of the Col shoot control samples were included in the linear model. The contrasts comparing “0.5 and 0 hrs” and “1 and 0.5 hrs” were then made. Genes with differential expression given a statistical threshold of an adjusted p-value < 0.05 for either comparison were separated into up- and down-regulated genes. The Wilcox test was performed to examine if the circadian amplitudes of differentially expressed CCGs and those of all CCGs are significantly different. The phase distributions of these up- and down-regulated CCGs were plotted in comparison to those of all CCGs to determine if any particular phase was overrepresented or underrepresented. Amplitudes and phases were determined by JTK_CYCLE using the meta time course data as described above.

## Supporting Information

Figure S1 A weighted Venn diagram indicating the overlap between the CCG lists identified from the separate Col and *rve8-1* datasets using JTK_CYCLE. The overlap is 44 and 49% of each data set. This level of agreement compares favorably to the one third overlap observed between two similar, independent data sets generated in Col [2], implying that the circadian rhythms in transcript levels between these two genotypes are unlikely to be significantly different.
(PDF)

Figure S2 Box plots presenting amplitudes of genes classified as clock-regulated in Col either by COSOPT alone (A) or JTK_CYCLE alone (B) and those of genes identified by both algorithms (JTK/COSOPT overlap). Genes only identified as cycling by one method have significantly lower amplitudes than those identified by both methods. The lower amplitudes indicate those genes are cycling less robustly and might explain why they are only found using one or the other method. The amplitudes are significantly different with p < 2.2e-16 (significance determined using Wilcox test).
(PDF)

Figure S3 A weighted Venn diagram presenting the overlap between CCGs identified in the meta time course data with all the transcripts present on Agronomics1 tiling array and the CCGs identified by Covington et al. [2] using the ATH1 array.
(PDF)

Figure S4 A thirty minute difference in circadian phase has profound effects on differential expression analysis. Differential expression analysis using a publicly available dataset of Col plants harvested at time 0.5 (corresponding to circadian time 3.5, herein defined “0.5 hr”) and 30 minutes later (herein defined “1 hr”) [29]. (A) A weighted Venn diagram presenting the proportion of clock-controlled genes (CCGs) among genes differentially expressed between the 1 and 0.5 hour samples (DE[1 hr - 0.5 hr]). (B) A box plot comparing the amplitudes of the differentially expressed CCGs (DE.cyc[1 h - 0.5 hr]) and those of all CCGs. The differentially expressed CCGs have on average significantly higher amplitude than all CCGs (p < 2.2e-16, Wilcox test). (C–D) The phase distributions of the CCGs classified as “up-regulated” or “down-regulated” between 1 and 0.5 hours are plotted alongside the observed phase distribution of all known CCGs (expected).
(PDF)

Figure S5 The promoters of the four evening-phased genes that are differentially expressed in *rve8-1* contain one or more EE (AAATATCT) or EE-like (AAAAATCT) sequences. The graph shown is the output of the SCOPE motif finder (http://genie.dartmouth.edu/scope/) [41].
(PDF)

Table S1 The circadian analysis by COSOPT. Agronomics1 microarray expression data for individual genes, normalized using RMA, and circadian parameters as determined using COSOPT. Phase indicates time of (day) peak expression, Beta indicates rhythmic amplitude, and pMMC.Beta indicates the agreement of the time series data with a cosine wave; the lower the pMMC.Beta value, the better the fit of the data to the best-matching cosine wave model tested. For our analyses, genes were defined as ‘expressed’ if more than four time points have expression levels higher than log_2_ (4.5). Genes with an pMMC.Beta value less than 0.05 were classified as circadian-regulated.
(CSV)

Table S2 The circadian analysis by JTK_CYCLE. Agronomics1 microarray expression data for individual genes, normalized using RMA, and circadian parameters as determined using JTK_CYCLE. PHASE indicates time of (day) peak expression, AMP indicates rhythmic amplitude, and ADJ.P (adjusted p value) indicates the agreement of the time series data with a cosine wave; the lower the ADJ.P value, the better the fit of the data to the best-matching cosine wave model tested. For our analyses, genes were defined as ‘expressed’ if they have at least four time points of expression levels greater than log_2_(4.5). Genes with an ADJ.P value less than 0.05 were classified as circadian-regulated.
(CSV)

Table S3 Primer sequences used in this study.
(XLS)
