# Supplementary figures and images for: Circadian Phase Has Profound Effects on Differential Expression Analysis
Source: PLoS One. 2012 Nov 20;7(11):e49853. doi: 10.1371/journal.pone.0049853 (PMC3502281; doi:10.1371/journal.pone.0049853)

**Fig. S1**

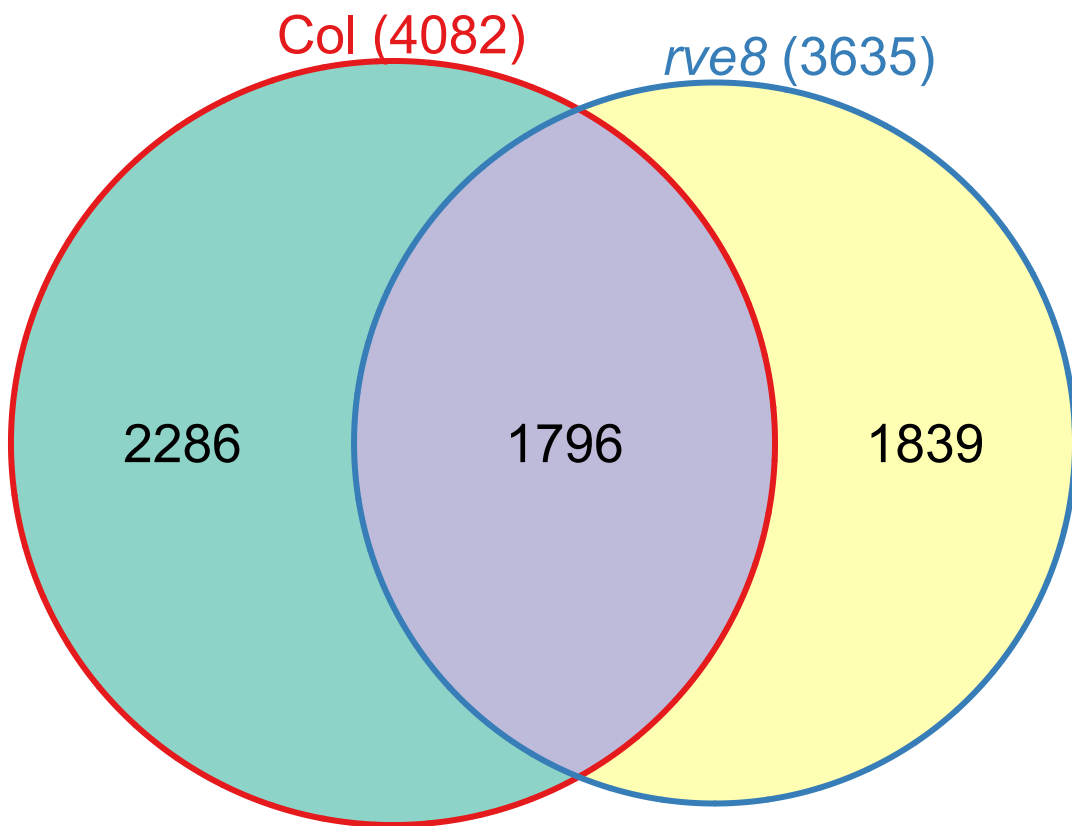

Supplement: Figure S1 — A weighted Venn diagram indicating the overlap between the CCG lists identified from the separate Col and rve8-1 datasets using JTK_CYCLE. The overlap is 44 and 49% of each data set. This level of agreement compares favorably to the one third overlap observed between two similar, independent data sets generated in Col [2], implying that the circadian rhythms in transcript levels between these two genotypes are unlikely to be significantly different. (PDF) [file pone.0049853.s001.pdf]

# Fig. S2

## A

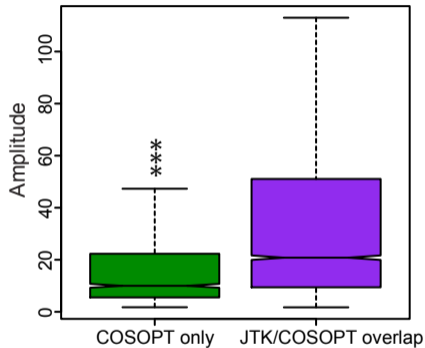

## B

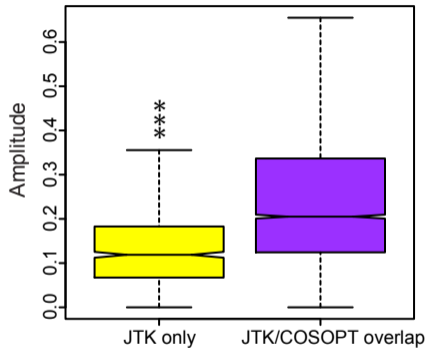

Supplement: Figure S2 — Box plots presenting amplitudes of genes classified as clock-regulated in Col either by COSOPT alone (A) or JTK_CYCLE alone (B) and those of genes identified by both algorithms (JTK/COSOPT overlap). Genes only identified as cycling by one method have significantly lower amplitudes than those identified by both methods. The lower amplitudes indicate those genes are cycling less robustly and might explain why they are only found using one or the other method. The amplitudes are significantly different with p < 2.2e-16 (significance determined using Wilcox test). (PDF) [file pone.0049853.s002.pdf]

# Fig. S3

Meta.C+J.intersect  
Agronomics1  
(5836)

Covington.meta  
ATH1 (3964)

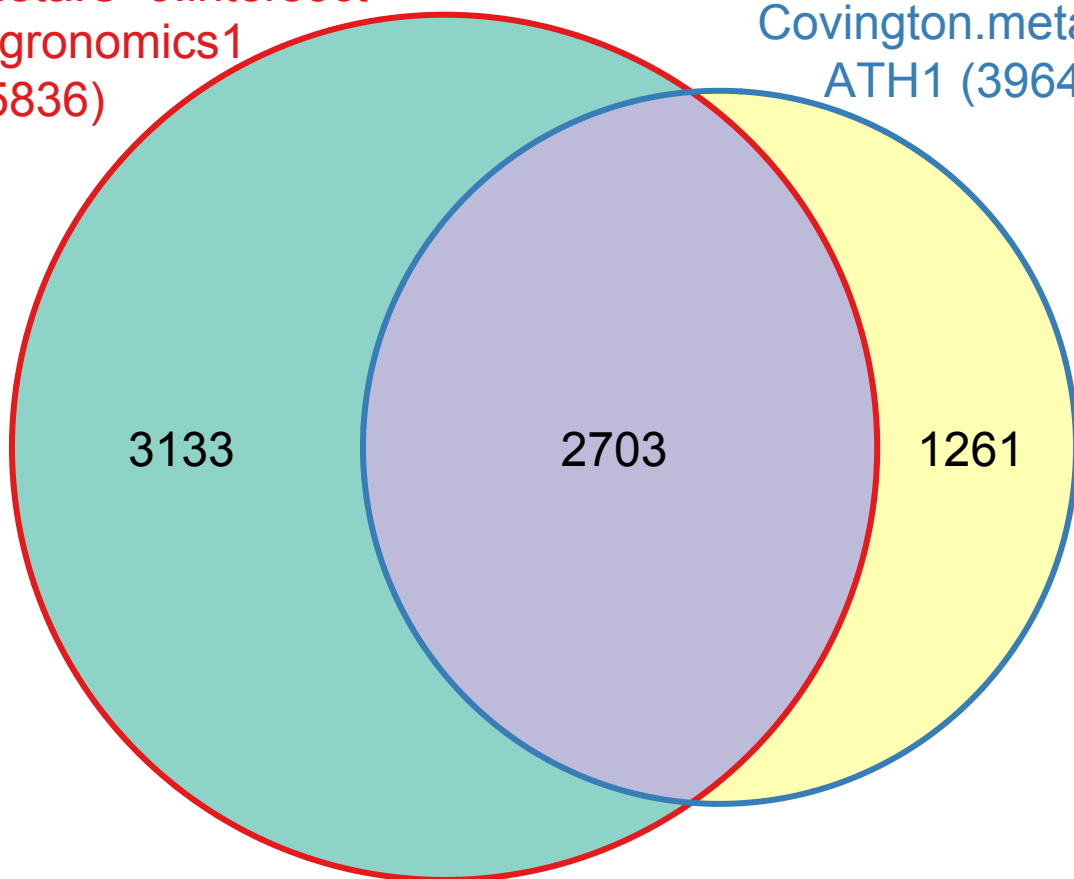

Supplement: Figure S3 — A weighted Venn diagram presenting the overlap between CCGs identified in the meta time course data with all the transcripts present on Agronomics1 tiling array and the CCGs identified by Covington et al. [2] using the ATH1 array. (PDF) [file pone.0049853.s003.pdf]

# Fig. S4

**A**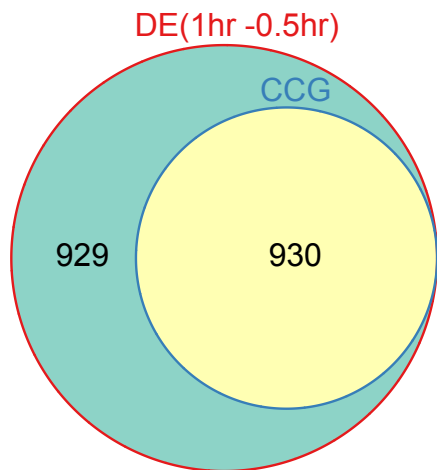**B**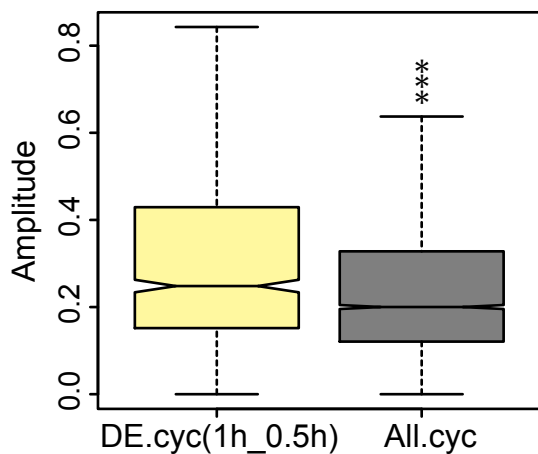**C**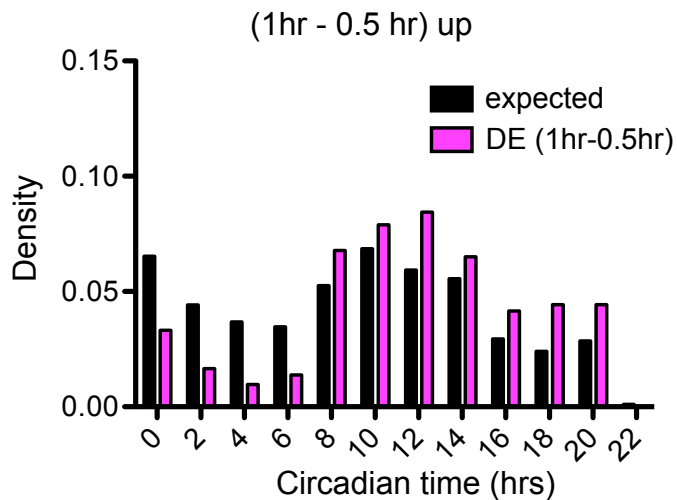**D**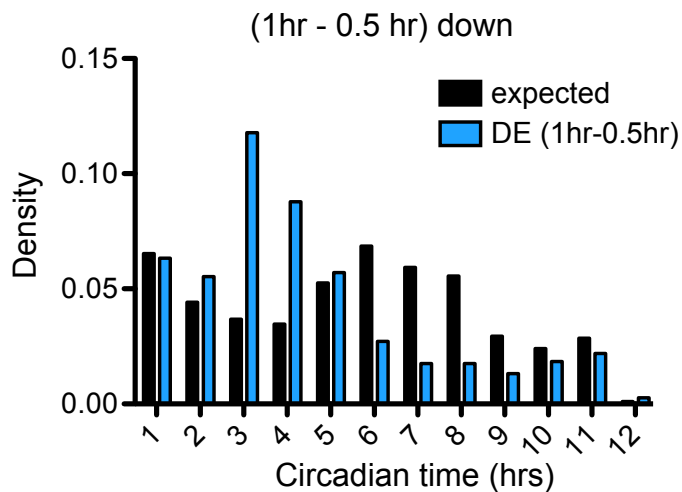

Supplement: Figure S4 — A thirty minute difference in circadian phase has profound effects on differential expression analysis. Differential expression analysis using a publicly available dataset of Col plants harvested at time 0.5 (corresponding to circadian time 3.5, herein defined “0.5 hr”) and 30 minutes later (herein defined “1 hr”) [29]. (A) A weighted Venn diagram presenting the proportion of clock-controlled genes (CCGs) among genes differentially expressed between the 1 and 0.5 hour samples (DE[1 hr - 0.5 hr]). (B) A box plot comparing the amplitudes of the differentially expressed CCGs (DE.cyc[1 h - 0.5 hr]) and those of all CCGs. The differentially expressed CCGs have on average significantly higher amplitude than all CCGs (p < 2.2e-16, Wilcox test). (C–D) The phase distributions of the CCGs classified as “up-regulated” or “down-regulated” between 1 and 0.5 hours are plotted alongside the observed phase distribution of all known CCGs (expected). (PDF) [file pone.0049853.s004.pdf]

Fig. S5

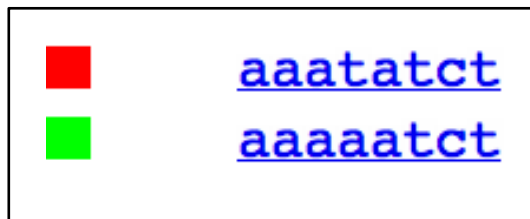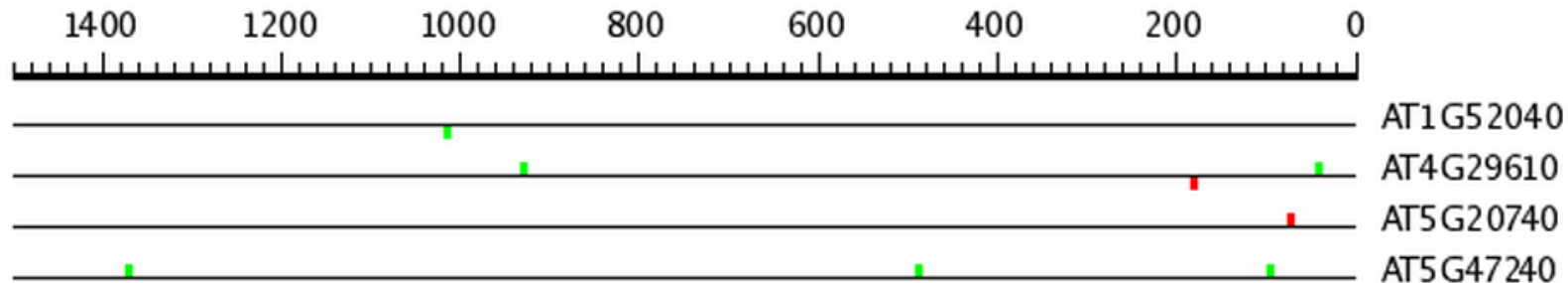

Supplement: Figure S5 — The promoters of the four evening-phased genes that are differentially expressed in rve8-1 contain one or more EE (AAATATCT) or EE-like (AAAAATCT) sequences. The graph shown is the output of the SCOPE motif finder (http://genie.dartmouth.edu/scope/) [41]. (PDF) [file pone.0049853.s005.pdf]
